# The genome of Clostridium difficile 5.3

**DOI:** 10.1186/1757-4749-6-4

**Published:** 2014-02-24

**Authors:** Aaron E Darling, Paul Worden, Toni A Chapman, Piklu Roy Chowdhury, Ian G Charles, Steven P Djordjevic

**Affiliations:** 1 ithree institute, University of Technology Sydney, Broadway Street, 2007 Ultimo, Australia; 2 NSW Department of Primary Industries, Elizabeth Macarthur Agricultural Institute, Woodbridge Road, 2568 Menangle, Australia

**Keywords:** Clostridium difficile 5.3, Genome, Sequencing

## Abstract

**Background:**

*Clostridium difficile* is the leading cause of infectious diarrhea in humans and responsible for large outbreaks of enteritis in neonatal pigs in both North America and Europe. Disease caused by *C. difficile* typically occurs during antibiotic therapy and its emergence over the past 40 years is linked with the widespread use of broad-spectrum antibiotics in both human and veterinary medicine.

**Results:**

We sequenced the genome of *Clostridium difficile* 5.3 using the Illumina Nextera XT and MiSeq technologies. Assembly of the sequence data reconstructed a 4,009,318 bp genome in 27 scaffolds with an N50 of 786 kbp. The genome has extensive similarity to other sequenced *C. difficile* genomes, but also has several genes that are potentially related to virulence and pathogenicity that are not present in the reference *C. difficile* strain.

**Conclusion:**

Genome sequencing of human and animal isolates is needed to understand the molecular events driving the emergence of *C. difficile* as a gastrointestinal pathogen of humans and food animals and to better define its zoonotic potential.

## Background

*Clostridium difficile* is a Gram-positive, spore-forming bacterium that over the last 40 years has emerged from obscurity to become a leading gastrointestinal pathogen in hospital environments [1]. The clinical features of disease range from non-haemorrhagic watery diarrhea to fatal episodes of pseudomembranous colitis and toxic megacolon. *C. difficile* disease is also community-acquired with incidence rates increasing [2]. In the past decade *C. difficile* has caused widespread outbreaks of neonatal diarrhea in piglets raising concerns that it may have a food-borne etiology. One porcine strain of *C. difficile* is frequently isolated from human cases of *C. difficile* disease in human in Europe, underscoring its zoonotic potential [3].

Non-toxigenic strains of *Clostridium difficile* have been isolated from patients with diarrhoeal disease [4]. Recent studies showing that nontoxigenic strains are capable of blocking colonisation and prevent disease caused by toxigenic strains indicates that much remains to be learned about virulence factors that play important roles in colonization of the gastrointestinal tract [5]. Here we report the sequence of nontoxigenic *C. difficile* strain 5.3 isolated from a patient with diarrhoea. The clinical presentation of this patient was of suspected *Clostridium difficile* infection. This was confirmed at the pathology laboratory with the isolation of only non-toxigenic *C. difficile*.

## Methods

### Isolation and DNA preparation

*Clostridium difficile* 5.3 originated from a faecal sample submitted from a female patient with the clinical symptomology of *C. difficile* infection. From the faecal sample 100 *μ*l of sample was added to cooked meat medium (TM0102 Oxoid Australia) and incubated anaerobically using the anoxomat system (MART Microbiology B.B., The Netherlands) at 37C for 24 hours. From here 200 *μ*l of the culture was transferred aseptically into a centrifuge tube and centrifuged at 10,000 rpm for 5 mins. The supernatant was discarded and the pellet resuspended in 1 ml of absolute ethanol and incubated at room temperature with periodic inversion for two hours.

A pellet was recovered by centrifuging at 10,000 rpm for 5 mins, the ethanol was discarded and the pellet resuspended in 100 *μ*l of brain heart infusion broth and spread plated onto *Clostridium difficile* selective agar (CC-BHIA + Taurocholate, PP2362 Oxoid Australia) and incubated anaerobically at 37 C for 24 hours. All plates were examined for colonies that morphologically represented *C. difficile*, from each plate colonies were selected and subcultured onto CC-BHIA + Taurocholate until pure cultures were achieved.

DNA was extracted from pure cultures by taking a single colony into 2 ml of brain heart infusion agar and incubating anaerobically at 37 C for upto 48 hours. The bacterial suspension was then vortexed and the DNA extracted using the DNeasy Blood and Tissue Kit (Qiagen, 69581) using the manufacturers instructions for the extraction of gram positive bacteria and the use of lysozyme.

### Genome sequencing

DNA was quantified using qubit flourimetry and 0.5 ng gDNA was used as input to the Illumina Nextera XT library preparation protocol. Tagmentation of Genomic DNA, and PCR amplification of tagged DNA were performed as per manufacturer’s instructions. However the “PCR Clean-Up” and “Library Normalization” steps were omitted and size selection was instead performed by running balanced and pooled samples in a one percent agarose gel and excising the 600 bp to 1200 bp region of interest. The DNA was then purified from the agarose using Promega’s Wizard SV Gel and PCR Clean-Up System. Finally, an Agilent 2100 Bioanalyzer, with a High Sensitivity DNA Kit, was used to quantitate the pooled DNA library before loading onto the MiSeq with 23 other multiplexed samples. Paired-end 300 nt reads were generated using MiSeq V3 chemistry.

### Assembly and annotation

The genome was assembled using a version of the A5 pipeline [6] called A5-miseq [7] that has been revised to process reads up to 500 nt long. Briefly, A5-miseq consists of five stages: (1) read quality filtering and error correction, (2) contig assembly, (3) permissive draft scaffolding, (4) misassembly detection, and (5) conservative scaffolding. The revised A5 pipeline uses a new version of idba_ud that uses read pairing information, and that has been modified to accept reads up to 500 nt long and to construct *de Bruijn* graphs with *k*-mers up to 500 nt. These modifications provide substantial improvements in assembly contiguity.

The genome was annotated with the RAST annotation system using FigFAM release 70 [8].

## Quality assurance

A5-miseq includes a quality checking step that detects putative misassemblies by identifying clusters of read pairs that map to disjoint locations in the assembled genome. This method did not detect any putative misassemblies.

## Initial findings

**Figure 1:**
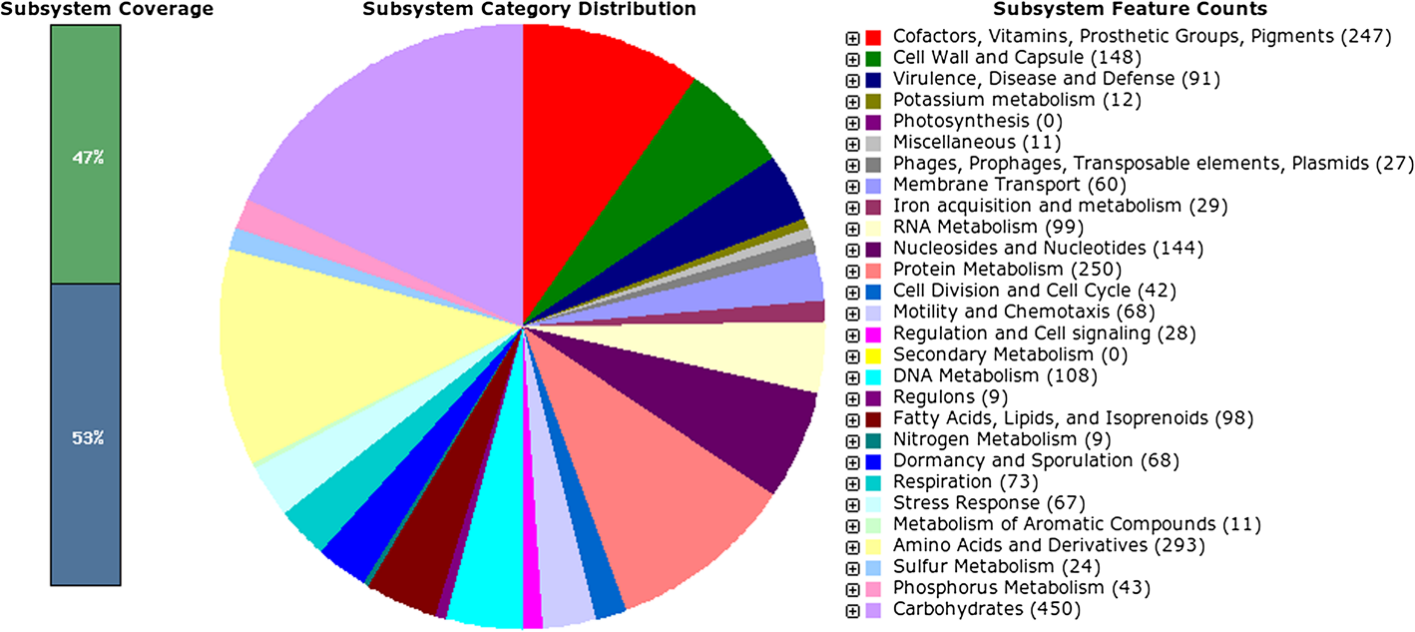
**Subsystems in *C. difficile *5.3.** 47% of the predicted CDS have been assigned to a subsystem in RAST. The functional category with the largest number of assigned CDS is carbohydrate metabolism, and *C. difficile* 5.3 has several genes in this functional category that are not present in the finished genome of *C. difficile* 630.

Sequencing generated 925,170 read pairs for a total of 555,102,000 nt that were assembled to reconstruct the 4,009,318 bp genome of *C. difficile* 5.3 in 27 scaffolds, with a scaffold N50 of 786 kbp and an N90 of 135 kbp. The raw (unfiltered) coverage is 138x, and after read filtering the assembly has a median depth of coverage of 60. The annotation of this assembly identified 3647 predicted CDS and 125 predicted RNA genes. 13 genes were identified as possibly missing from the assembly by the RAST system. The overall functional profile of the genome is shown in Figure 1. We conducted a phylogenetic analysis of *C. difficile* 5.3 using the PhyloSift software [9] to identify the most closely related organism with an available reference genome, using a phylogenetic placement [10] on a tree constructed from a set of 37 universally conserved genes. The resulting analysis assigned a posterior probability of 100% for *C. difficile* 5.3 diverging on the same lineage as *Clostridium difficile* 6503. Because *Clostridium difficile* 6503 has a draft-quality genome we used the closely related genome of *Clostridium difficile* 630 as a reference for further comparative analysis.

**Figure 2:**
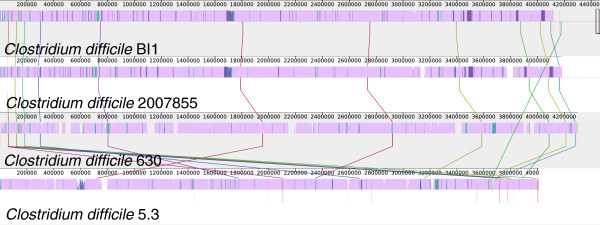
**Genome alignment of *C. difficile* BI1, *C. difficile* 2007855, *C. difficile* 630, and *C. difficile* 5.3.** A comparison of the four genomes as visualized by the Mauve software is shown. The four genomes are free from large-scale rearrangement, and exhibit high levels of sequence identity throughout their genomes, with a number of differentially conserved regions. Regions conserved among all genomes are shown in the color mauve, regions shared among subsets of the genomes are in different colors, regions unique to any particular genome are white.

The scaffolds of *C. difficile* 5.3 were reordered to match the order in the finished genome of the closely related strain *C. difficile* 630 using the Mauve Contig Mover [11]. After reordering we find the genomes are free from large-scale rearrangement. Rearrangement breakpoints for some small translocated regions occur at sites annotated with phage-related gene functions, suggesting that bacteriophage may have lysogenized at different locations in each strain. The genome of *C. difficile* 5.3 was aligned to those of *C. difficile* BI1, *C. difficile* 2007855, and *C. difficile* 630 to characterize the extent of gene content sharing and genomic rearrangement. A visualization of the genome comparison produced by the Mauve software is shown in Figure 2. From this figure we can see that the majority of the genome is conserved among all four isolates, although some strain-specific regions exist.

Comparison of the gene content between *C. difficile* 5.3 and the finished *C. difficile* 630 reference genome identified 105 annotated gene functions predicted to be present only in *C. difficile* 5.3. In particular, genes related to metal resistance, bacitracin resistance, colicin resistance, drug efflux, and several phage-related genes are present only in *C. difficile* 5.3. These genes may contribute to the pathogenic phenotype of *C. difficile* 5.3. A full list of these genes has been provided in Additional file 1.

## Future directions

Improved efficiency of the clinical genomics pipeline will enable fine-scale epidemiological monitoring of *Clostridium difficile* outbreaks.

## Availability of supporting data

The draft genome assembly has been submitted to NCBI and assigned accession PRJNA232267. Genome annotations are available from the RAST web server under accession 6666666.54620 when logged in with username guest, password guest. The Illumina sequence reads have been deposited to the Short Read Archive and under accession SRX396630.

## Abbreviations

RAST: Rapid annotation using subsystem technology; A5: Andrew and Aaron’; s Awesome Assembly; gDNA: genomic DNA; nt: nucleotides.

## Competing interests

The authors declare that they have no competing interests.

## Authors’ contributions

TAC provided the DNA. PW constructed Illumina libraries and sequenced them. AED conducted the assembly, analysis, and data deposition. AED, TAC, SPD, and PW wrote the paper. IGC and PRC provided general direction. All authors read and approved the final manuscript.

## Supplementary Material

Additional file 1 Listing of gene functions present in Clostridium difficile 5.3 that are not found in the reference strain Clostridium difficile 630. Click here for file
